# A protocol for a multidisciplinary early intervention during chemotherapy to improve dietary management behavior in breast cancer patients: a two-arm, single-center randomized controlled trial

**DOI:** 10.1186/s12885-024-12623-w

**Published:** 2024-07-18

**Authors:** Han Tang, Wei Zhang, Haiyan Shen, Haili Tang, Min Cai, Tao Wang, Pei Yan, Liang Li, Yan Wang, Huadong Zhao, Lei Shang

**Affiliations:** 1 Department of Health Statistics, School of Public Health, The Fourth Military Medical University, Xi’an, 710032 China; 2 Department of Clinical Nursing, School of Nursing, The Fourth Military Medical University, Xi’an, 710032 China; 3 Department of Orthopedics 1, Sichuan Provincial People’s Hospital, Chengdu, 610072 China; 4 Department of General Surgery, the Second Affiliated Hospital, Air Force Medical University, Xi’an, 710038 China; 5 Department of Psychiatry, the First Affiliated Hospital, Air Force Medical University, Xi’an, 710032 China; 6 The Medical Department, the First Affiliated Hospital, Air Force Medical University, Xi’an, 710032 China; 7 Department of Operation Room, the First Affiliated Hospital, Air Force Medical University, Xi’an, 710032 China; 8 Department of Thoracic Surgery, the Second Affiliated Hospital, Air Force Medical University, Xi’an, 710038 China

**Keywords:** Breast cancer, Chemotherapy, Diet, Health management behavior, Nutrition, Quality of life

## Abstract

**Background:**

Adverse reactions are prone to occur in the early stage of chemotherapy and can negatively affect the dietary intake and nutritional status of breast cancer (BC) patients. Consequently, they need to participate in health self-management and lifestyle promotion programs. Early multidisciplinary interventions aim to enhance dietary management behavior and quality of life in chemotherapy-treated BC patients.

**Methods:**

This single-blinded, single-center, randomized controlled trial will include 88 females who have not yet started the early or middle stage of the chemotherapy cycle. A random number table will be used randomly assign females to the intervention group or usual group at a 1:1 ratio. The intervention elements are based on the theoretical guidance of the Integrated Theory of Health Behavior Change (ITHBC). A multidisciplinary team (MDT) comprising oncologists, dietitians, nurses, traditional Chinese medicine (TCM) practitioners, and psychologists will provide the intervention. Intervention sessions will be conducted once a week for 8 weeks, beginning in the early or middle stage of the chemotherapy cycle and continuing through admission and a home-based interval chemotherapy period. The intervention includes face-to-face discussions, online meetings, WeChat messaging, and telephone calls. The themes target adverse reactions, dietary information and habits, self-care self-efficacy, treatment self-regulation, dietary supplement and TCM use, social support, weight management, and outcome expectations. The primary outcome is dietary management behavior measured by the Dietary Management Behavior Questionnaire (DMBQ). Secondary outcomes are self-care self-efficacy assessed by the Strategies Used by People to Promote Health (SUPPH); quality of life measured by the Functional Assessment of Cancer Therapy-Breast (FACT-B); and body mass index (BMI) measured by an electronic meter. All participants will be assessed at baseline and immediately, 1 month, 3 months, 6 months, and 12 months after the intervention.

**Discussion:**

Early dietary intervention is needed, as diet is one of the most common health self-management behaviors influenced by chemotherapy. Early multidisciplinary interventions may provide a foundation for dietary self-management and improve nutritional status in the survival period.

**Trial registration:**

This intervention protocol was registered with the Chinese Clinical Trials Registry (ChiCTR2300076503, October 10, 2023).

**Supplementary Information:**

The online version contains supplementary material available at 10.1186/s12885-024-12623-w.

## Background

According to the latest global cancer epidemiological data released by the World Health Organization [1], there were approximately 2.3 million new cases of breast cancer (BC) in 2020, accounting for 11.7% of all new cancer cases worldwide (excluding basal cell carcinoma). Furthermore, there were approximately 400,000 new cases of BC among Chinese women in 2020, and this number is expected to increase further [2]. Although the incidence of BC is high, several studies [2, 3] have confirmed that the 5-year relative survival rate can reach 40.5% or more, and the proportion of survivors is also increasing steadily. This is primarily attributed to improvements in diagnosis and treatment.

At present, chemotherapy is one of the most common and essential therapies for treating BC. The vast majority of BC patients receive chemotherapy after diagnosis. However, a series of side effects caused by chemotherapy, such as nausea, vomiting, loss of appetite, taste disorders, and gastrointestinal symptoms, can seriously affect the dietary intake and nutritional status of BC patients. This further exacerbates weight gain and muscle strength loss and has a profound negative impact on the quality of life and psychological state of BC survivors [4]. Possible reasons include the following: first, drug-related toxicity leads to muscle depletion due to decreased protein anabolism and proatrophic mechanisms, resulting in decreased skeletal muscle mass and increased fat mass; second, chemotherapy can cause ovarian failure, which affects hormone levels and is also associated with subsequent weight gain; third, chemotherapy-related fatigue can reduce daily physical activity and energy expenditure at rest, resulting in weight gain; and finally, chemotherapy can lead to changes in taste and smell and can cause changes in eating habits, which can affect weight [5]. Diet is one of the most important lifestyle factors and is considered a self-management health behavior involving physiological, psychological, environmental, social, and other factors [6]. Some researchers [6, 7] have proposed early nutritional interventions at the beginning or in the middle of chemotherapy, but specific evidence about BC patients needs to be supplemented. Therefore, BC patients’ dietary management behavior (DMB) during chemotherapy should be considered.

There are few existing studies about dietary management interventions for cancer patients during chemotherapy, and the available studies lack specificity. First, regarding the intervention population, most studies [8–10] have focused on BC survivors who have completed all adjuvant therapy. However, many females report the most severe side effects during chemotherapy. They do not know how to cope with these side effects and receive little support during treatment [11–13]. Thus, early dietary intervention is needed. Regarding intervention content, most cancer patients are thought to be provided with professional nutritional supplements [14, 15]. The nutritional status of most cancer patients does not reach severe pathological malnutrition. Instead, they can feed themselves orally and desire increased knowledge about nutrition and dietary precautions. Implementing DMB interventions is highly beneficial. These interventions can stimulate patients’ sense of autonomy, cultivate their nutritional management ability, provide a basis for the implementation of home-based rehabilitation plans after chemotherapy, and ultimately improve their life expectancy and quality of life [5].

Additionally, diet and nutrition promotion are usually framed as the work of oncologists and dietitians. Nevertheless, diet is an integral part of one’s lifestyle and a health management behavior that includes physiological, psychological, environmental, societal, and cultural elements [16, 17]. In China, cancer patients also desire advice on food therapy or integrative medicine from traditional Chinese medicine (TCM) practitioners [18, 19]. A multidisciplinary team (MDT) comprising oncologists, dietitians, clinical nurses, TCM practitioners, and psychologists may be suitable for providing diet management interventions.

Many previous interventions were designed without theoretical guidance, and only a few intervention studies considered social cognition theory [9, 10, 20, 21], cognitive behavior theory [22], or World or American Cancer Guidelines [8, 23]. Most studies have focused on weight management, nutrient intake, and exercise among BC survivors. Chemotherapy side effects can disrupt patients’ regular diets and require self-management behaviors to transition to a healthy diet. By integrating the core concepts of a variety of commonly used theoretical models that affect health behavior change (such as self-regulation theory, health behavior change theory, and social facilitation theory) and through empirical research, a patient-centered and dynamic middle domain theory model called the ‘Integrated Theory of Health Behavior Change (ITHBC) was developed. The ITHBC states that ‘change’ is a dynamic and circular process, emphasizing that establishing behaviors requires the combined effects of multiple aspects such as improving patient cognition, enhancing self-management skills, and creating a supportive environment [24]. According to this theory, the factors related to health behavior change include knowledge and beliefs, self-regulation, and social support; the resulting health behavior is the proximal outcome, and the health status is the distal outcome. Based on the ITHBC, one study [25] used corresponding variables to evaluate the factors influencing weight management in overweight postpartum women. The results showed that knowledge and beliefs about self-efficacy, outcome expectations, self-regulation, and social support could affect weight management behavior. However, this theory does not apply to the DMB of BC patients during chemotherapy. Given that the ITHBC is a comprehensive health behavior change theory and provides clear elements that may relate to health behavior, we used the concepts and factors of this theory as the theoretical basis for the construction of the content of this intervention. The outcome stated in this theory was used as the outcome indicator of this intervention to verify the effect. In previous studies, the control group received standard health education from the department at admission. According to the ITHBC, the expected benefits of the intervention include better DMB and physiological and psychological health conditions.

This study is the first multidisciplinary early intervention during the beginning and middle of the chemotherapy cycle to improve DMB in BC patients, focusing mainly on health self-management behavior and lifestyle promotion based on the theoretical guidance of the ITHBC. This intervention may save large amounts of medical resources and significantly enhance patients’ confidence in their health management participation, which can help them maintain their nutritional status and prepare them for later recovery during the survival period. In addition, multidisciplinary interventions enable management by health professionals while simultaneously promoting patient self-management, ensuring continuous and comprehensive healthcare services. The aim of this randomized controlled trial (RCT) is to explore the feasibility of a multidisciplinary early dietary management intervention provided during chemotherapy from the perspective of health behavior change theory and its effectiveness on DMB (proximal outcome) and quality of life (distal outcome) in female BC patients.

## Methods/design

This manuscript followed the Standard Protocol Items: Recommendations for Interventional Trials (SPIRIT) guidelines [26] (Supplementary materials 1). Ethics approval was granted by the ethics committee of the Second Affiliated Hospital of the Fourth Military Medical University (number: K202305-41). This trial was registered with the Chinese Clinical Trials Registry (ChiCTR2300076503; October 10, 2023).

### Study design

**Fig. 1:**
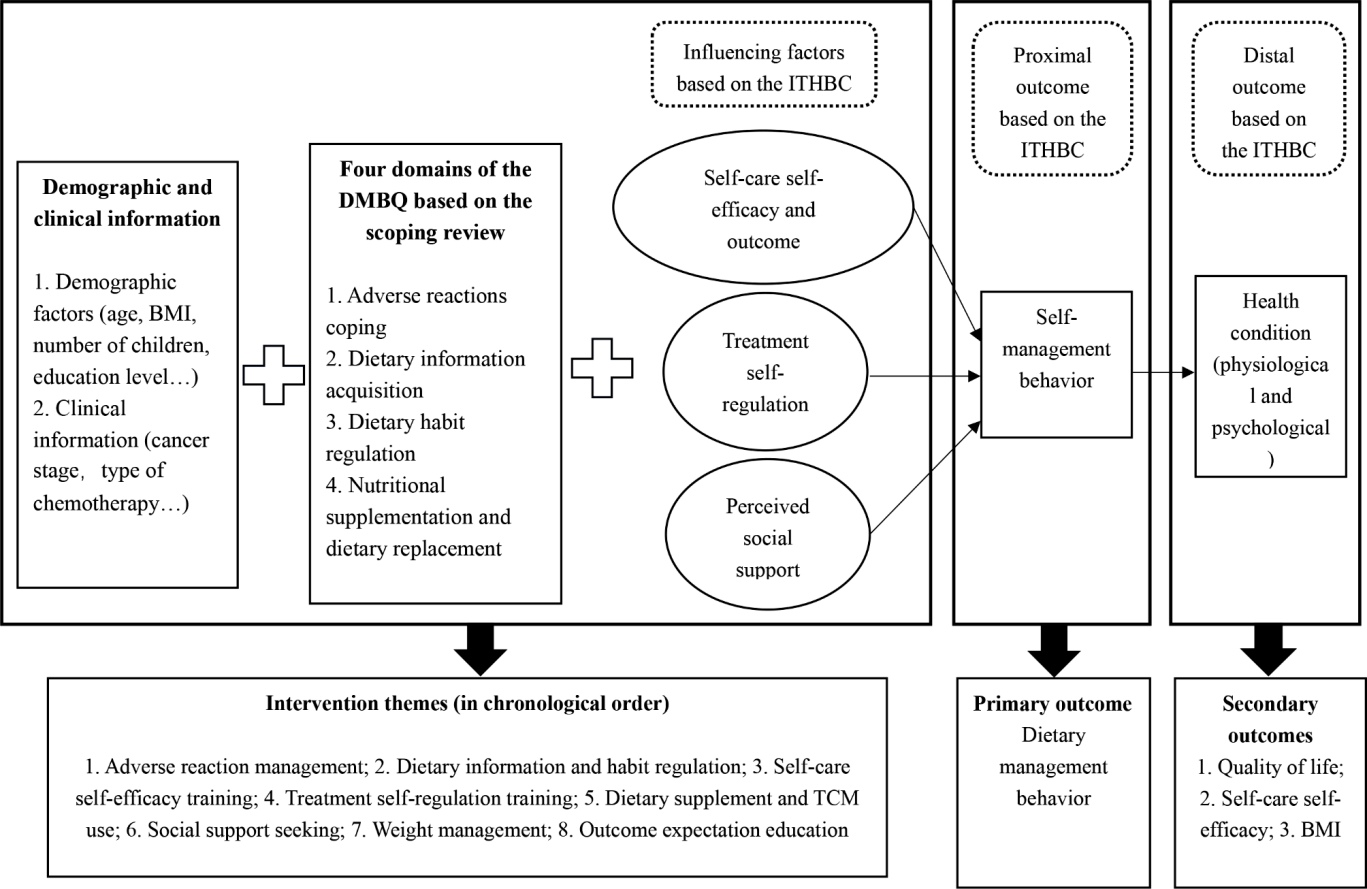
Conceptual frameworks for the multidisciplinary early intervention. BMI: body mass index; DMBQ: Dietary Management Behavior Questionnaire for breast cancer patients treated with chemotherapy; ITHBC: Integrated Theory of Health Behavior Change; TCM: traditional Chinese medicine

**Fig. 2:**
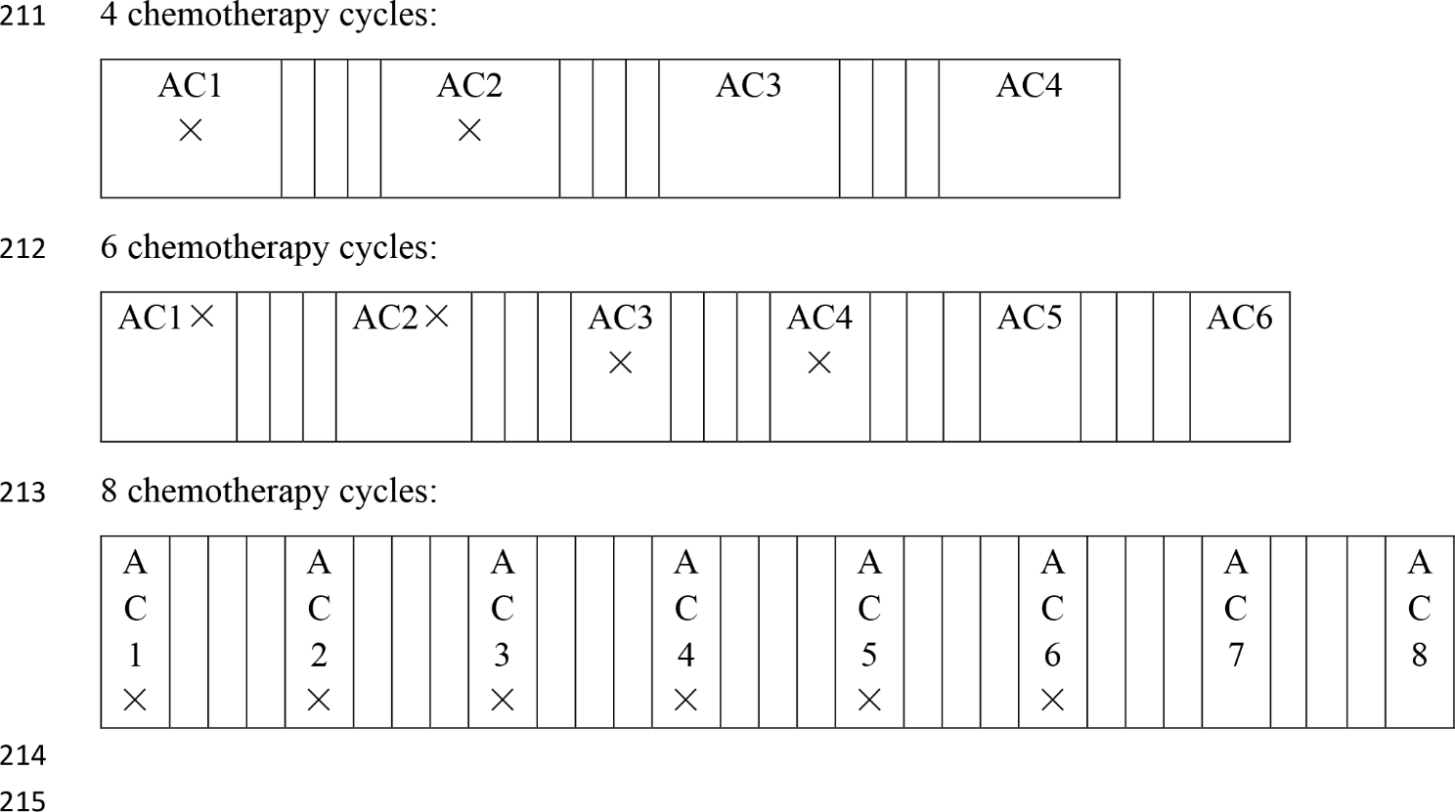
Initiation of the multidisciplinary early intervention for patients with different numbers of chemotherapy cycles. AC: each admission chemotherapy ×: possible time to start the intervention (early and intermediate stages of the whole chemotherapy cycle); a blank space: a one-week intermission of chemotherapy at home

**Fig. 3:**
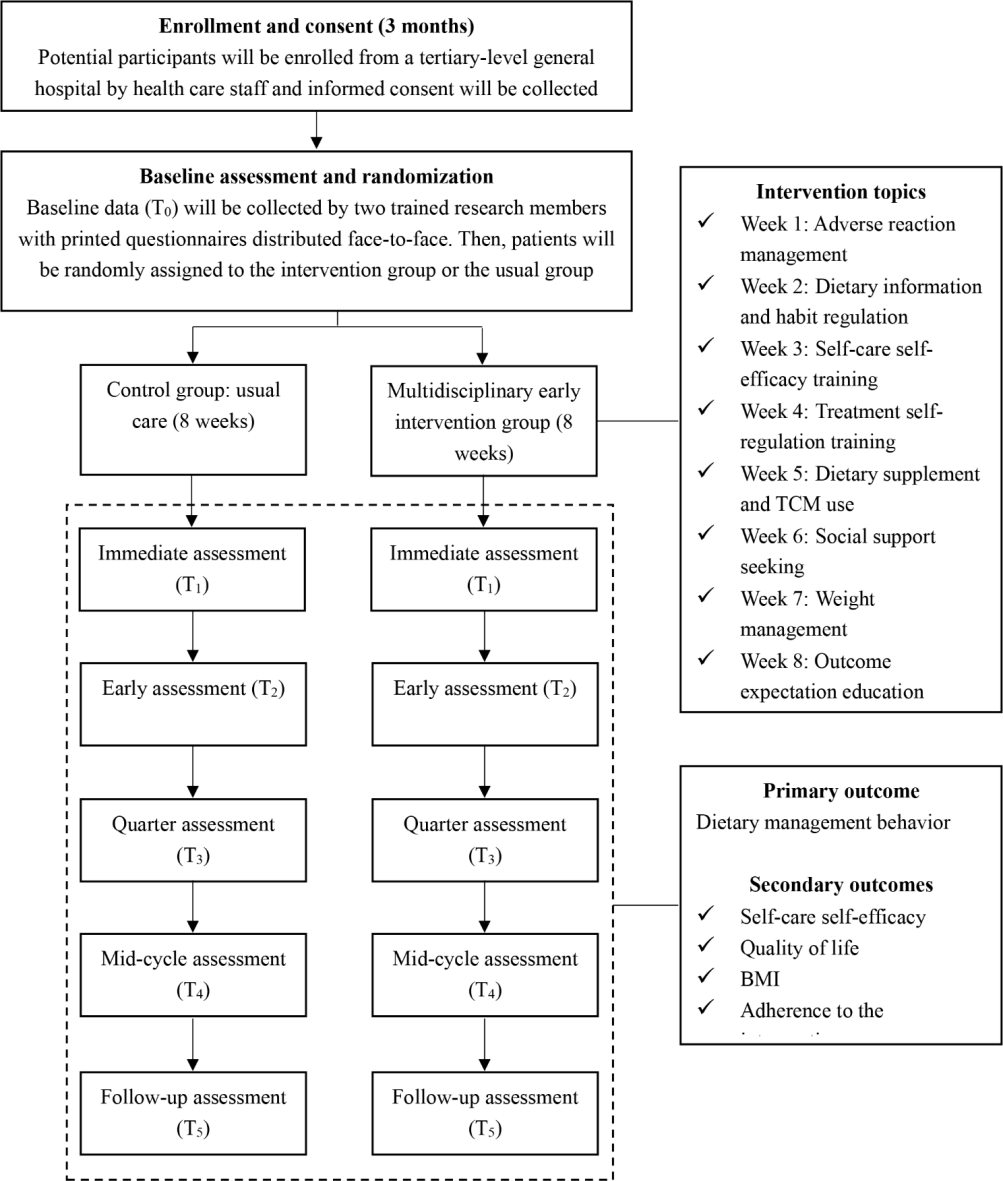
The flow chart of the intervention process in this RCT. T_0_: before the intervention; T_1_: immediately after the intervention; T_2_: 1 month after the intervention; T_3_: 3 months after the intervention; T_4_: 6 months after the intervention; T_5_: 12 months after the intervention. BMI: body mass index. RCT: randomized controlled trial

A prospective 8-week, two-arm, single-center, single-blinded parallel-group RCT with a pretest and follow-up will be conducted by an MDT. Female patients who are diagnosed with BC and waiting to start chemotherapy or who are in the early or middle stage of chemotherapy will be randomized to receive either usual dietary care or the multidisciplinary early dietary management intervention. The participants in the intervention group will receive the multidisciplinary early intervention measures constructed based on the ITHBC. In contrast, the control group will receive only the usual cancer dietary care education at admission and education manuals based on the Dietary Guidelines for Chinese Residents, the World or American Guidelines, etc. The intervention consists of the following eight themes, with sessions provide once per week: adverse reaction coping, dietary information and habit regulation, self-care self-efficacy training, treatment self-regulation training, dietary supplement and TCM use, social support seeking, weight management, and outcome expectation education. Detailed information is provided in Fig. 1. Eight intervention sessions will be provided throughout admission for chemotherapy and a home-based interval chemotherapy period. This intervention will be initiated during the early and middle stages of chemotherapy. Standard chemotherapy cycles include 4, 6, and 8 cycles. For example, for patients receiving 4 chemotherapy cycles, the first intervention session will be provided during the first or second admission for chemotherapy. For patients receiving 6 cycles, the first intervention session will be provided during the first, second, third, or fourth admission. For patients receiving 8 cycles, the first intervention session will be provided during the first, second, third, fourth, fifth or sixth admission. The duration of intervention sessions during different chemotherapy cycles is shown in Fig. 2. In addition to the baseline measurements, follow-up measurements were taken immediately after the end of the intervention (T_1_) and at 1 (T_2_), 3 (T_3_), 6 (T_4_) and 12 months (T_5_) after the intervention. T_1_ was measured immediately after the end of the eighth intervention session. T_2_, T_3_, T_4_ and T_5_ will be measured 30, 90, 180 and 360 days after the T_1_ measurement date, respectively. The intervention process is shown in Fig. 3. The same time points for follow-up in the intervention group will be also used for the control group.

### Recruitment and procedures

The potential participants will be enrolled from a tertiary-level general hospital in Shaanxi, China. The hospital is an essential medical resource in Northwest China, and the affiliated hospital of a well-known medical university with a profound history and accumulation in China. It has a large cancer center and provides continuous care from the inpatient to home-based interval chemotherapy periods for more than 3000 BC patients annually. We will contact participants in two main ways, the first being through active contact. Second, posters will be placed in waiting rooms and examination rooms at the oncology department or breast surgery department of this hospital, advertisements will be posted on the department’s official website, WeChat public account, video platform, etc., or word of mouth will be used. Interested women will be asked to contact research members via phone, email, or WeChat for detailed information. Regarding the second approach, researchers will learn about potential participants who meet the requirements through recommendations from health care staff in the department. After obtaining informed consent, the research team members will obtain the patients’ names and numbers, contact the patients to introduce the specific purpose and process of this study, and then ask them about their willingness to participate.

### Participant eligibility

The inclusion criteria for female patients are as follows: (1) aged ≥ 18 years, have been diagnosed with malignant BC by pathological examination and are aware of their disease and treatment; (2) have an order for chemotherapy and a regimen that has been established by oncologists according to their condition; (3) will be undergoing or receiving adjuvant or neoadjuvant chemotherapy; (4) have not yet initiated the early or middle stage of chemotherapy (for patients with a total of 4 chemotherapy cycles who have not yet had their first or second admission for chemotherapy; for a total of 6 cycles who had not yet had her the first, second, third or fourth admission chemotherapy; for a total of 8 cycles who had not yet had her the first, second, third, fourth or fifth or sixth admission for chemotherapy); (5) are able to orally feed themselves independently; (6) have no need for additional enteral or parenteral nutrition support after evaluation by the oncologists and dieticians; (7) can speak and understand Chinese, are aware of this research and agree to participate voluntarily; and (8) have good physical and mental status to support the progress of the intervention. The exclusion criteria are as follows: (1) end-stage cancer, such as systemic lymph node metastasis, a predicted survival of less than 6 months; (2) malignant tumors at other sites; (3) a plan to become pregnant, current pregnancy or becoming pregnant within 6 months after delivery; (4) family disagreement with study participation; (5) a history of psychiatric illness, severe cognitive impairment or severe visual, hearing or language impairment; (6) a poor nutritional status or pathological eating disorder, morbid obesity, etc.; and (7) participation in similar research.

### Sample size

G power software will be used to calculate the sample size of the study, and ANOVA and a priori power analysis were selected [27]. Because there is little relevant literature and most multidisciplinary health management interventions do not report detailed sample size calculation parameters, we adopted system default parameters, including an effect size of 0.25, a power of 0.95, and an α of 0.05. After calculation, the total sample size was 72, with 36 participants in each group. Considering a 20% loss-to-follow-up rate, we obtained a final sample of 44 participants per group.

### Randomization

We seek to enroll 88 eligible BC patients during a 3-month recruitment period. The patients will be provided identifiers based on the enrollment order and will be randomly assigned to the intervention or usual groups. A random number Table [28] will be used to divide the 88 patients into two groups at a 1:1 ratio. We will start from the numbers in row 5 and column 6 of the random number table, and random numbers will be selected in turn from left to right. If the exact number is encountered, it will be skipped, and the following number will be skipped until 88 different numbers are collected. All the numbers will then be arranged from small to large, with patients with 44 smaller numbers assigned to the control group and those with the 44 larger numbers assigned to the intervention group. The random sequence will be unknown to the remaining research members except the statistician.

### Blinding

This is a multidisciplinary intervention on dietary management for BC patients, and the BC patients and intervention providers will know the group assignments. Because three face-to-face training sessions for MDT members will be conducted before the intervention is implemented and patients need to receive eight diet-related health education sessions, blinding patients and intervention providers may be challenging. Thus, a single-blinded design will be used, in which the statisticians who analyze the data are not aware of the group assignments.

### Intervention

The intervention protocol was designed according to the Dietary Guidelines for Chinese Residents, National Comprehensive Cancer Network (NCCN) clinical practice guidelines for BC, and the World or American Nutrition Guidelines. The average daily diet should include more than 12 kinds of food or more than 25 kinds of food with reasonable collocation, including cereals and tubers, vegetables and fruits, livestock, poultry, fish, eggs, milk and legumes; however, a balanced dietary pattern of cereals should be followed. According to the Chinese food Pagoda, daily intake of 1500–1700 ml of plain drinking water, less than 5 g of salt, and 25–30 g of oil will be encouraged. Daily intake of cereals should be limited to 200 to 300 g. Daily consumption of vegetable and fruit should be 300–500 g and 200–350 g, respectively. A total of 120–200 g of animal-based foods, including one egg per day, should be consumed. The amount of milk and dairy products should be 300–500 g, and that of soy and nuts should be 25–35 g per day. Conversely, the consumption of excessive amounts of sugar-sweetened beverages, fatty, smoked and preserved meats, and prepackaged foods should be avoided, as these food groups are not beneficial for health [29, 30]. In addition to clinical insights, cognitive, psychological, and behavioral perspectives are equally crucial for DMB. Therefore, we also conducted a scoping review of the performance of DMB interventions and the key elements, adding to the exploration of influencing factors based on the ITHBC. Later, expert consultation methods and patient pilot tests were used to revise and determine the final version of the intervention.

**Fig. 4:**
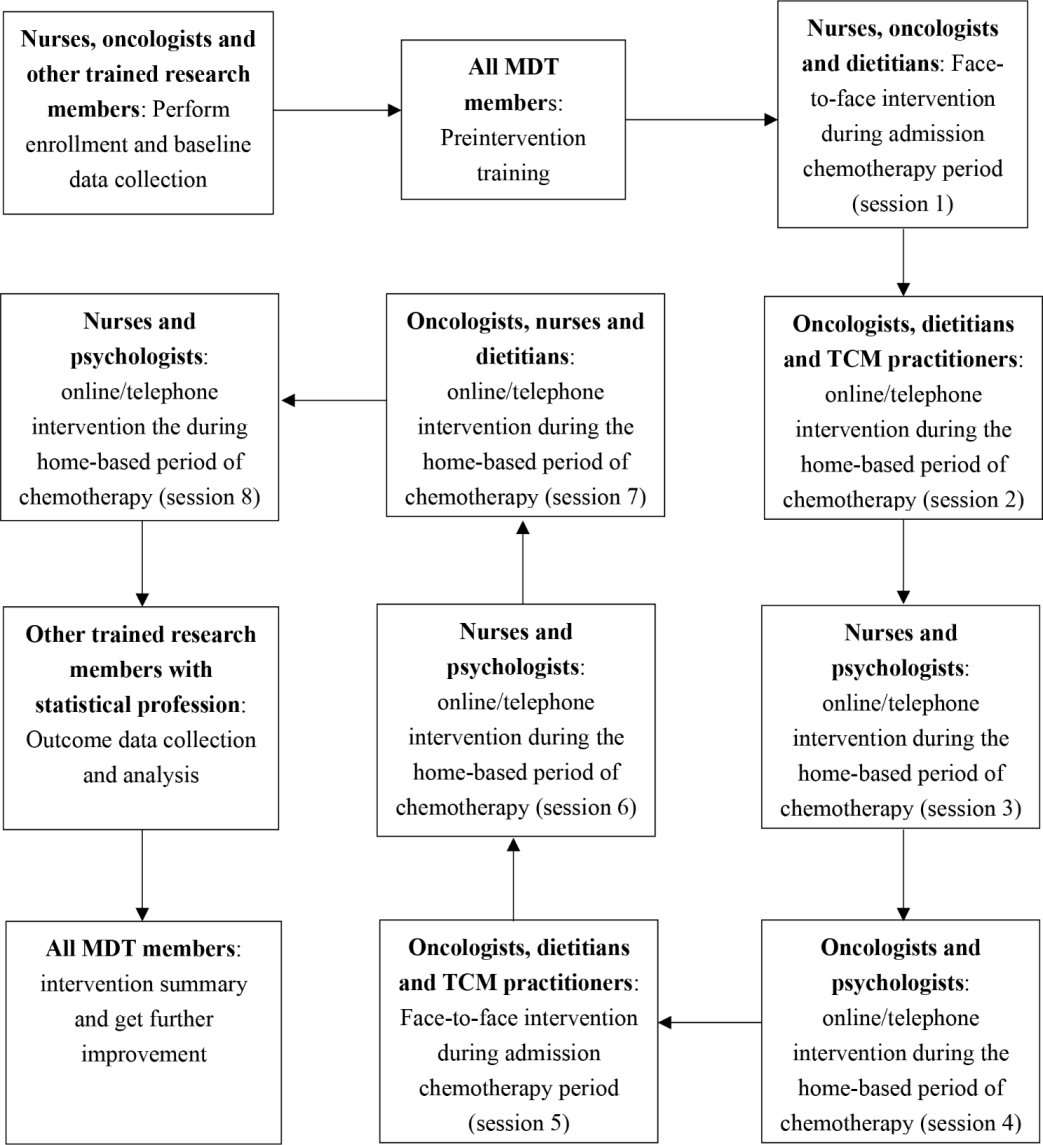
The flow chart and core tasks of the team members in this multidisciplinary early intervention. MDT: multidisciplinary team; TCM: traditional Chinese medicine

Before the intervention, it will be necessary to establish a MDT team and clarify the division of work to ensure the quality of the intervention. We reviewed the literature on multidisciplinary interventions as comprehensively as possible and combined the characteristics of DMB in BC patients; the MDT providing this intervention will comprise oncologists, clinical nurses, dietitians, TCM practitioners, psychologists, and other research members. The detailed core tasks of team members are illustrated in Fig. 4.

### Division of work and roles of MDT members in the multidisciplinary early intervention

1. Oncologists: This group will comprise 4 oncologists from the oncology department or breast surgery department of the hospital. Before the intervention, one oncologist will be responsible for contacting potential participants and assisting with patient recruitment. Another oncologist will provide professional information about the disease and chemotherapy during the intervention. The remaining two oncologists will provide information on healthy weight and adequate food intake according to the patients’ BMI categories and nutritional status and help them to develop individualized management goals.
2. Clinical nurses: This group will comprise a total of 4 nurses. One nurse will assist with patient recruitment before the intervention. During the intervention, one nurse will be responsible for delivering knowledge about coping with adverse reactions and strategies related to dietary self-management, another nurse will provide psychological support, and the last nurse will assist oncologists in formulating targeted diet management goals for patients with different conditions.
3. Dietitian: One dietitian will provide professional dietary and nutritional information and demonstrations, such as common dietary misunderstandings of BC patients during chemotherapy (excessive intake of supplements, replacing normal intake of health care products with three meals, eating only vegetables and no meat, supplementing nutrition by drinking more soup, etc.), the relationship between hormones and diet, correct dietary intake (assessed by a dietitian considering patient age, sex, energy and nutrient needs, physical activity level and the presence of specific pathologies), and scientific methods of weight management (according to the patient’s physical characteristics, a scientific program will be provided to monitor and record food and water intake on the day of weight management). The core elements include reducing caloric intake, increasing the liquid diet appropriately, and controlling hunger.
4. TCM practitioners: One TCM practitioner in the group will convey scientific information on nutritional supplements and dietary replacement from the perspective of TCM, as well as food therapy practices and precautions, which are of great concern to Chinese patients.
5. Psychologist: One psychologist will be responsible for providing professional knowledge, including enhancing self-management confidence, providing treatment self-regulation education, training in social support seeking, and outcome expectation training. Moreover, patients will be led in playing games such as “identifying automatic thoughts, testing hypotheses, examining evidence, identifying alternative thoughts, defending counsel, and challenging wrong, extreme thoughts”.
6. Other research members: Two trained statistical professionals will be primarily responsible for patient recruitment and randomization before the intervention. The other two team members will collect all the data from baseline to follow-up. A statistician will also analyze the data.
7. Team initiator and coordinator: The initiator of this intervention is the oncology department direct, who has rich clinical experience and professional scientific research ability. The coordinator is a nurse with a doctoral degree who organizes and coordinates each MDT meeting, reports to patients, and performs preintervention preparations.

### Team training before the intervention

The researchers will draft training materials based on the Dietary Guidelines for Chinese Residents and guidelines from the Chinese Society of Nutritional Oncology. Then, the contents will be determined after a complete discussion with experts in various fields. After the intervention protocol is designed and three weeks before the formal implementation, three face-to-face training sessions will be conducted in the department meeting room; one training session will be provided per week, lasting approximately 2–3 h. During the first session, the oncologists and nurses will study the training materials and then teach other MDT members basic knowledge of BC and chemotherapy, coping methods for adverse reactions after chemotherapy, the relationship between hormones and diet among female BC patients, and the average weight standard for BC patients. Next, the researcher will introduce the necessity and feasibility of this multidisciplinary early intervention to improve patients’ DMB. During the second training, the dietitian and TCM practitioner will teach the team members how to carry out the targeted DMB intervention during the admission and home-based chemotherapy periods, how to guide patients in choosing an appropriate dietary nutrition supplement, the scientific methods of weight management, etc. Then, the researcher will provide a specific explanation of the flow and contents of the intervention. During the last session, the psychologist will introduce the necessity of maintaining a healthy mental state for cancer patients undergoing chemotherapy; analyze the standard sources, types, and factors related to pressure in BC patients; demonstrate strategies to help patients achieve stress relief; clarify the key points; and train patients to improve their self-management efficacy, self-regulation, and social support. Then, the researcher will further explain the frequency, time, division of work, and considerations of the intervention.

The training will include lectures, interactive workshops, case discussions, clinical practice, and scenario simulations. After the training, 5 BC patients treated with chemotherapy will be invited to participate in case analysis and scenario simulation, and the accuracy of the evaluation results will be discussed with the healthcare staff of the oncology department.

### Implementation of the multidisciplinary early intervention

A total of 8 intervention sessions will be conducted once a week, each lasting approximately 1.5–2 h. The intervention will begin during the early or middle stage of the whole chemotherapy cycle and continue through admission and a home-based interval chemotherapy period. During the first chemotherapy admission, patients will receive face-to-face group or individual interventions. During the home-based interval chemotherapy period, online meetings, WeChat messaging, or telephone calls will be adopted. The core elements of the 8 intervention sessions are listed as follows:

1. Intervention session 1 (topic: adverse reaction management): After the participants receive chemotherapy during admission and 1 day before hospital discharge, the MDT members will introduce themselves and the describe intervention process face-to-face, and patients will become familiar with each other. This session will include group interventions, group discussions and lectures: (1) patients will share their experiences with chemotherapy and diet; the group will discuss their own disease and chemotherapy experiences and diet change trajectory; (2) a nurse will show a professional video from the China Central Television (CCTV) “Health Road” program about BC, chemotherapy and diet; (3) oncologists, nurses, and dietitians will introduce the trend of common adverse reactions and the corresponding dietary self-management tips during different chemotherapy periods, including early, middle, and late-term chemotherapy or chemotherapy during admission or the interval period; and (4) oncologists will clarify the boundaries between dietary self-management and medication management when adverse reactions occur.
2. Intervention session 2 (topic: dietary information and habit regulation): A combination of online group and individual intervention sessions will be conducted during the first week of the home-based interval chemotherapy period after session 1, which will include the following: (1) oncologists and dietitians will hold an online group Tencent conference to introduce authoritative sources of dietary information for cancer patients, such as WeChat public accounts, short videos, guidelines, and distributed information in the form of electronic materials; (2) dietitians and TCM practitioners will focus on correct dietary habits and misunderstandings for individuals with different demographic characteristics during telephone calls or WeChat messaging, including discussing the relationship between dietary intake and hormone levels for hormone receptor-positive patients, providing nutrition and weight management clinics and food therapy for patients with higher family income or abnormal weight; and (3) dietitians will demonstrate methods of dietary intake self-assessment by online video presentations, using simple and intuitive representations such as “a handful, a fist, 1 g or 2 g of quantitative salt spoons”.
3. Intervention session 3 (topic: self-care self-efficacy training): The online group session during the second week of interval chemotherapy after session 1 will include the following: (1) nurses will guide patients to disclose their concerns about participating in their diet self-management; (2) the psychologist will lead “evidence check” and “advocate” sessions: patients will be asked to write evidence for and against their participation in dietary self-management on two post-it notes and then play the advocate, attacking the evidence against participation; and (3) nurses will provide diet option comparison cards to show patients the pros and cons of different dietary habits and encourage all patients to choose and share the reasons.
4. Intervention session 4 (topic: treatment self-regulation training): Online group and individual sessions will be conducted during the third week of interval chemotherapy after session 1, which will include the following: (1) Through WeChat, the psychologist will send patients an assessment questionnaire linked to treatment self-regulation. According to the results, the patients will be divided into four groups: the autonomous motivation, introjected regulation, external regulation, and amotivation groups [31]. Then, the psychologist will explain definitions, content, and performance to the same online group Tencent conference; (2) the psychologist will explain the relationship between mood and diet and then use “identifying automatic thoughts” and “testing hypotheses” to determine the cognitive reasons for the lack of motivation among patients in the amotivation group in the process of participating in diet self-management and demonstrate stress relief techniques, such as deep breathing and listening to soothing music; and (3) nurses will invite patients in the autonomous motivation and introjected regulation groups to share their diet self-management experiences and the goals achieved.
5. Intervention session 5 (topic: dietary supplement and TCM use): The next admission chemotherapy period after the session, which will include a face-to-face group lecture and discussion one day before hospital discharge, will include the following steps: (1) dietitians and TCM practitioners will use videos, charts, and paper materials to explain the concept, indications, and types of dietary nutritional supplements and replacement therapy (which can be divided into two categories: edible nutritional supplements as well as complementary and alternative therapies, such as moxibustion, massage, and aromatherapy); (2) through clinical practice and scenario simulations, using pictures or natural objects, oncologists and dietitians will guide patients in analyzing and choosing dietary supplements and replacement therapies according to their conditions; and (3) TCM practitioners will describe the content of food therapy that Chinese patients are interested in, popularize scientific knowledge and correct misunderstandings.
6. Intervention session 6 (topic: social support seeking): Online group and individual sessions will be performed during the first week of home-based interval chemotherapy after session 5, which will include the following: (1) warm small things to share and express gratitude: nurses will encourage patients to write down the names of people who prepare their meals and living arrangements during chemotherapy through an online group Tencent conference and prepare a sentence, video or gift for them. (2) The psychologist will provide targeted dietary management tips related to emotion and psychology depending on the working status of patients via WeChat or telephone. For employed females, dietary advice will be provided to their families or colleagues before they return to work. Nonemployed individuals will be encouraged to share their thoughts and preferences regarding their diet with friends or neighbors who would like to help take care of them. (3) Nurses will encourage patients to share how others can help improve their diet during chemotherapy and how they seek help.
7. Intervention session 7 (topic: weight management): Online group and individual sessions will be provided during the second week of the interval chemotherapy period after session 5, which includes (1) oncologists informing patients that being overweight is a risk factor and showing patients how to correctly measure their body weight via an online video, calculate their body mass index (BMI) and determine their BMI classification. (2) Dietitians will introduce the criteria and ranges of daily caloric intake for patients with different BMI classification via WeChat or telephone. For example, overweight and obese patients will be taught to read ingredient lists and recipes. Patients with a BMI below the normal range will be taught to choose foods rich in high-quality fat (such as omega 3 fatty acids and monounsaturated fatty acids) and protein [29, 30]. Repeated operations and repetitions will be used to test the effect. (3) Nurses will show patients and their caregivers how to exercise moderately and record daily calories using apps or memos.
8. Intervention session 8 (topic: outcome expectation education): An online group session during the third week of interval chemotherapy after session 5 will include the following: (1) the psychologist will introduce the concept, content, necessity, classification and function of the outcome expectation related to dietary self-management to patients by an online group Tencent conference; (2) Nurses will encourage patients to write down their expectations for diet self-management on post-it notes and classify them into immediate or future goals; (3) The psychologist will use the “five-column chart” of alternative thinking to ask patients to share their views on the outcome expectations and determine the negative cognition of achieving the goal; (4) Through scenario and case studies, the psychologist will lead patients to analyze their own cognition on two outcome expectations of what points may be right or wrong, encourage them to create new goals and teach them how to stick to long-term dietary self-management monitoring; and (5) MDT members will summarize and share the gains and suggestions of this intervention with patients.

### Criteria for discontinuing or modifying allocated interventions

According to the criteria for discontinuing allocated interventions, the intervention will be discontinued when the participants ask to withdraw or when their health conditions worsen. In addition, the parts of the intervention that patients think are too intense to adhere to or to be unreasonable will be revised. During the intervention, we will also carefully record any suggestions given by patients and make adjustments after complete discussion.

### Intervention fidelity

A nurse not involved in this trial will evaluate the fidelity of the intervention using the Modified Fidelity Checklist (MFC) [32]. This checklist comprises the adherence (18 items) and competence subscales (14 items) and a cover page about the intervention setting, times, number of participants in each group, etc. For the former subscale, all items are coded “yes” or “no,” with comments in the subsequent column. The first three items and the last item belong to the knowledge survey pretest and posttest, respectively, to determine the knowledge benefits related to the multidisciplinary early diet management intervention. The remaining 14 items reflect the content of this intervention. The latter subscale assesses whether the intervention promotes increased effort to facilitate patients’ efforts to achieve their intervention goals. Items are coded as “1 = skill rarely or never demonstrated, 2 = skill sometimes/occasionally demonstrated, and 3 = skill consistently demonstrated”, with additional qualitative feedback on the skill for an item.

### Control group: usual care

The patients assigned to the control group will receive standard admission health education from the department. The main contents include the following: After the BC patients are admitted to the hospital, the doctor will ask about the patient’s condition and provide basic BC disease information. Nurses will distribute health education manuals, such as adverse reactions and coping methods related to chemotherapy. In addition, oncologists and nurses will provide clinical group lectures to explain the relationship between chemotherapy and diet, dietary information and strategies (mostly from the Dietary Guidelines for Chinese Residents, the World or American Guide, etc.), the necessity of weight management, etc. Health professionals will use face-to-face communication, question answering, demonstrations, and other methods during admission for chemotherapy to solve dietary problems. Patients who need food therapy will be evaluated by doctors and provided with relevant knowledge, precautions, indications and contraindications. During the home-based chemotherapy interval, telephone calls, WeChat messaging, online conferences, emails, and other forms of assistance will be provided to BC patients as needed. As is usual practice, no specific or individual recommendations will be provided.

### Expert and patient involvement

After the detailed intervention protocol is developed, 10–20 experts and patients will be invited to make suggestions regarding the intervention. The researchers will conduct expert consultations by email or face-to-face. According to the professional fields involved, combining the principles of representativeness and authority, the selection criteria for the experts are as follows: (1) are oncologists, breast specialist nurses, clinical dietitians, psychologists, TCM practitioners, chronic disease health management experts, etc.; (2) have 5 years of work experience or more; (3) have an intermediate professional title or above; (4) have a bachelor’s degree or above in the nursing field and a master’s degree or above in medicine or other fields; and (5) volunteer to participate in the consultation and have rich professional knowledge and a rigorous academic attitude. Immediately after the completion of each expert consultation, the intervention protocol will be modified. After the completion of the modification, expert consultation will be conducted until all of the experts have finished. Then, several BC patients will be conveniently sampled after obtaining and reviewing their electronic medical records. We will first obtain their consent and determine their opinions about the intervention by asking questions such as “Can you tell us about your dietary experiences during chemotherapy? And “What changes or difficulties have you had with your diet since starting chemotherapy?” In parallel, patients will be invited to a multidisciplinary meeting to share their thoughts and participate in developing their management intervention content. After the intervention is modified, we will conduct a pilot study to assess the participation burden and duration. Based on the feedback and performance, the final intervention for which we plan to implement during the RCT will be identified. Patients will also be encouraged to recommend the intervention to the public or other patients.

### Outcome and measurements

We determined the intervention outcomes based on the theoretical guidance of the ITHBC, which states that patients’ knowledge and beliefs, self-regulation, and social support may influence their health behavior (proximal outcome) and health status (distal outcome). The primary outcome of this intervention is dietary management behavior. The secondary outcomes are psychological and physiological health status. The effect of the intervention will be compared with that of the control, both as a primary and secondary outcome. Each indicator is an independent metric.

### Primary outcome

**Dietary management behavior.** The patient-reported Dietary Management Behavior Questionnaire (DMBQ) will be used to assess dietary management behavior in BC patients treated with chemotherapy. Our group members previously developed this questionnaire in 2023 based on the theoretical guidance of the ITHBC and the Capacity, Opportunity, Motivation-Behavior Model (COM-B) [33]. After conducting a scoping review to construct an item pool, three rounds of a multicenter survey were completed, including item selection and evaluation of the reliability, validation, and discriminative ability of the formal verified version. This tool comprises 4 domains and 22 items, including the adverse reaction coping (4 items), dietary information acquisition (5 items), dietary habit regulation (8 items), nutritional supplements, and dietary replacement (5 items) domains. The items are rated on a 5-point Likert scale ranging from ‘strongly disagree’ (1 point) to ‘strongly agree’ (5 points), with higher scores indicating better DMB. The structure was tested among 760 Chinese BC patients treated with chemotherapy from three hospitals. The total cumulative variance contribution rate of the 4 domains and the 22-item questionnaire was 62.67%, the Cronbach’s α coefficient was 0.908, the split-half reliability coefficient was 0.833, and the test-retest reliability coefficient was 0.938. Moreover, there were significant differences in the average scores of each dimension among patients with different ages, education levels, BMIs, menopausal statuses, and histological stages (all *P* < 0.05), and these differences were reflected in different pairwise comparison groups, indicating good reliability, validation and discriminative ability in BC populations.

### Secondary outcomes

#### Self-care self-efficacy

The ‘Strategies Used by People to Promote Health (SUPPH)’ assessment tool will be used to evaluate BC patients’ self-care self-efficacy. The self-reported SUPPH has been widely used among chronic disease patients. It was translated into Chinese and introduced in China by Huijuan Qian in 2011 [34] and was verified among cancer patients. The Chinese version includes 3 domains and 28 items, including the positive attitude (15 items), stress reduction (10 items), and decision making (3 items) domains. The items are rated on a 5-point Likert scale ranging from ‘very little confidence’ (1 point) to ‘very confident’ (5 points), with higher scores indicating greater confidence in health self-management. The three domains’ split-half reliability coefficients and the Chinese version’s overall scale were 0.80 ~ 0.94, and the Cronbach’s α coefficient was 0.85 ~ 0.97.

#### Quality of life

We will adopt the Chinese version of the Functional Assessment of Cancer Therapy-Breast (FACT-B, version 4) introduced by Chonghua Wan [35] in 2002 to measure BC patients’ quality of life. This self-reported instrument has been widely used worldwide, with 5 domains and 36 items, including the physical well-being (PWB, 7 items), social/family well-being (SWB, 7 items), emotional well-being (EWB, 6 items), functional well-being (FWB, 7 items) and additional concerns (AC, 9 items) domains. The items are rated on 5-point Likert scale ranging from ‘not at all’ (0 points) to ‘very much’ (4 points), with higher scores indicating a better quality of life. The test-retest reliability of the five domains from the Chinese version, namely, the PWB, SWB, EWB, FWB, and AC domains, and the overall scale was 0.82 ~ 0.89, and the Cronbach’s α coefficient for the five domains was 0.61 ~ 0.84.

### Other outcomes

#### Demographic and clinical information

Self-designed demographic and clinical factor questionnaires will be used to collect this information. To ensure the representativeness and comprehensiveness of the demographic and clinical factors of BC patients as much as possible, we referred to guidelines and previous literature related to diet, nutrition, lifestyle, and self-health management in BC patients treated with chemotherapy. After discussion among the research team members, the questionnaire will be used to collect demographic (age, BMI, marital status, number of children, educational level, per capita monthly household income, living conditions, work status, and menopausal status) and clinical information (time of diagnosis, unilateral or bilateral BC status, cancer stage and chemotherapy type).

#### BMI

To minimize bias, we will provide each patient with an electronic weighing scale of the same make, model, and batch. The patients will be told to perform measurements in the morning after defecation and urination, wearing only underwear. Considering the limitations of the patients’ ages and educational levels as well as to improve operability, direct input of data by the patients will be avoided. Patients will be asked to weigh themselves at 7–8 am, and the data will be uploaded to their bound app and sent to the researchers via a screenshot. Screenshots of each measurement for each patient will then be recorded, checked by two researchers, entered into statistical software, and automatically saved in the cloud. The screenshot will show the specific date, time, and weight value. Height will be recorded after being measured using the hospital’s anthropometer at the patient’s initial admission. BMI will be calculated directly by using the formula in the statistical software.

#### Adherence to the intervention

The MDT members will track and summarize patient personnel participation at the end of each week’s intervention session. Each participant’s adherence to the intervention will be defined as the number of times the participant attends a complete session compared to the number of prescribed sessions, including completing the assigned tasks and actively interacting with MDT members to provide feedback. Specifically, we defined the nonadherence criteria as (1) being late or leaving early (more than one hour) during the intervention; (2) failing to complete the assigned task more than 3 times; (3) no communication or interaction with MDT members for more than half a month; (4) failing to follow up more than 3 times; and (5) being absent during more than 2 sessions.

### Data collection

Two other professionally trained researchers will collect the data with a statistical professional. Printed questionnaires will be distributed to participants face-to-face at baseline (T_0_). The questionnaires will be distributed and collected on the spot, and the members will check for missing or unclear information. In addition, an electronic questionnaire collection platform will be used immediately (T_1_) and 1 month (T_2_), 3 months (T_3_), 6 months (T4) and 12 months (T_5_) after the intervention. An answer will be required for each item. According to the preliminary pilot test, the average completion time is approximately 10–20 min. The completion time will be recorded in the background of the system. Patients who take less than 6 min or more than 30 min will be asked to complete the questionnaire again or excluded if the questionnaire is invalid. Patients will receive a reward of RMB 300 if they complete all assessments promptly. At each measurement, the entire questionnaire will include the demographic and clinical information questionnaires, the DMBQ, the Chinese version of the SUPPH and FACT-B, and BMI measurement.

### Statistical analysis

We will use IBM SPSS Statistics 21.0 software to analyze the statistical data. Descriptive statistics, such as the number of patients with different ages, education levels, cancer stages and types of chemotherapy, as well as the percentage of patients in different groups relative to the total number of patients, will be calculated for different groups of demographic and clinical information. The mean and standard deviation will be used to compare the DMBQ total and dimensions scores, the SUPPH and FACT-B scores, and BMI. To compare the baseline balance between the two groups, two independent sample *t* tests, chi-square tests or Mann‒Whitney U tests, will be used, depending on whether the data distribution satisfies homogeneity of variance. The Shapiro‒Wilk test will be used to verify whether the scores of the two groups at different time points follow a normal distribution. If the data are balanced, repeated ANOVA will be adopted. Otherwise, we will use a generalized estimating equation (GEE) to analyze the intervention’s time, group, and interaction effects [36]. For all the above tests, *P* < 0.05 will indicate a statistically significant difference, the α level will be 0.05, and the tests will be two-sided. We will use intention-to-treat (ITT) analysis to minimize bias for sensitivity analysis [37]. The results of the last measurement of the patients who drop out of the study will be taken as the effect, which assumes that they complete the intervention, and the analysis will be reconducted with the GEE. Furthermore, missing data will be imputed with the mean values of the characteristics or with the help of multiple imputation methods if they are random and unrelated to the research elements.

### Data management and monitoring

The personal and outcome information of all participants will remain confidential. During the data entry and analysis phase, each patient will be given a serial number, in the order of completion. A nurse from the research team will be responsible for data storage, and only she will have access to the data. All the raw data will be stored in electronic files, and each access requires the insertion of an authorized USB with a password, which guarantees the confidentiality of the data as much as possible. Moreover, a data and safety monitoring board (DSMB) will ensure data and quality assurance for the intervention. The DSMB consists of three experts, excluding those involved in this trial. Excluding trial investigators, the DSMB will hold online/face-to-face meetings semiannually to assess and review the trial protocol, interim reports, recruitment process and numbers, sample characteristics, outcome measures, and any adverse events. In addition to following the standard procedures for reporting adverse events, patients will be regularly followed up by telephone or home visits to assess the occurrence of adverse events. The assessment scope includes events that may harm a patient’s health or prolong treatment, aggravate a patient’s condition, cause medical disputes, and cause unnecessary financial losses for a patient. Once adverse events are identified, they will first be reported to the research team and clinical department and then submitted to the hospital’s scientific research and administrative department after submitting a written report. Apart from bottom-up reporting, the intervention will be subject to any form of monitoring by the DSMB. The primary role of the DSMB is to ensure that the trial is conducted scientifically and safely and to provide suggestions for the trial based on existing problems at different stages. Reports generated by the DSMB will also be sent to the research team and the ethics committee.

## Discussion

Chemotherapy can not only lead to adverse gastrointestinal symptoms [38, 39] but also cause negative chemosensory changes and food perceptions in BC patients [40, 41]. Both factors influence females’ energy intake and nutritional status [42]. Previous studies [13, 43–45] have shown that in the early stage of chemotherapy, a series of adverse reactions, such as nausea, vomiting, and dysgeusia, occur, immediately leading to daily dietary intake disorders, which may manifest as increased or decreased appetite, increased intake of high-calorie foods, or picky eating, followed by body weight and body composition changes. Another study [46] reported that the dietary intake of BC patients was the same as that of healthy women before chemotherapy. However, the average daily energy intake of the BC group during chemotherapy was 214 kcal less than that of the control group. As diet is a long-term and essential lifestyle component, and changes in dietary habits caused by chemotherapy will continue to occur during recovery, advanced stages, and survival [47]. Limited studies [48, 49] have shown that BC patients who actively manage chemotherapy-related symptoms and actively collect nutritional information may recover better and have better quality of life. This suggests the importance and necessity of early dietary management interventions during chemotherapy.

However, most existing interventions and guidelines target BC survivors who have completed all adjuvant treatments or assess qualitative and quantitative diet composition in terms of nutrients, and the elements that do not improve adherence are relatively limited. Regarding research concepts and principles, in our intervention, we regarded diet as a comprehensive behavior consisting of physical, psychological, social, cultural, and environmental factors. We explored the content with the guidance of health behavior change theory. Given the Chinese dietary culture background and the clinical characteristics of females with BC, we referred to national and international dietary recommendation guidelines. We included TCM and food therapy points that Chinese patients are interested in. Early dietary management interventions will be provided during chemotherapy, and professional TCM practitioners will be invited to provide scientific knowledge and correct common misunderstandings; these practitioners will try to combine localization and professionalism to the greatest extent possible in the intervention program. For protocol design research methods, many existing intervention protocols are guided by theory. We previously conducted a scoping review about the performance and elements of DMB in BC patients undergoing chemotherapy and summarized themes with the guidance of the COM-B. After determining the main conceptual framework of DMB, we selected potential factors by the ITHBC and maintained them as intervention elements that would significantly influence DMB. The intervention elements are comprehensive and include psychology (self-care self-efficacy, treatment self-regulation, social support, and outcome expectations), information and physiology (adverse reaction coping, dietary information and habits, and weight management), nutrition, and culture (dietary supplement and TCM use). Finally, theory was also used to determine the proximal and distal outcomes of the intervention. Additionally, we extended the measurement range from immediately to 12 months after the intervention to the explicit effect of the trend of change over time. For the intervention provider and form, we organized an MDT involving oncologists, nurses, dietitians, psychologists, TCM practitioners, and statisticians to provide an 8-week early intervention for BC patients from admission to home-based interval chemotherapy, which may provide a foundation for dietary self-management for the late-stage chemotherapy and survival periods. However, since this is a preliminary study protocol that mainly tests the feasibility of the intervention program, the study will be limited by factors such as manpower and material resources. In addition, this study focuses on the perspective of health behavior change, which should be added to food records in the future in addition to psychological and social outcome indicators. These indicators could represent useful tools for evaluating dietary habits, intervention adherence and the association with the primary outcome (DMB) of the study.

In conclusion, this study will focus on the DMB of BC patients during chemotherapy. This study will establish an MDT to provide an early and comprehensive intervention to improve the nutritional status of BC patients and provide a basis for health management and quality of life for survival. A rigorous design will be used, guided by appropriate theory. Participants will be randomly assigned to two groups, and baseline characteristics will be assessed to determine any significant differences. The main questionnaire that will be used was developed with scientific steps and strict verification, which further ensures the rigor of the study design.

### Electronic supplementary material

Below is the link to the electronic supplementary material.

Supplementary Material 1

## Data Availability

No datasets were generated or analysed during the current study.
